# Improving counterfactual reasoning with kernelised dynamic mixing models

**DOI:** 10.1371/journal.pone.0205839

**Published:** 2018-11-12

**Authors:** Sonali Parbhoo, Omer Gottesman, Andrew Slavin Ross, Matthieu Komorowski, Aldo Faisal, Isabella Bon, Volker Roth, Finale Doshi-Velez

**Affiliations:** 1 Department of Mathematics and Informatics, University of Basel, Basel, Switzerland; 2 School of Engineering and Applied Sciences, Harvard University, Cambridge, Massachussets, United States of America; 3 Department of Bioengineering, Imperial College, London, United Kingdom; 4 Department of Experimental, Diagnostic and Specialty Medicine, University of Bologna, Bologna, Italy; Universita degli Studi di Pisa, ITALY

## Abstract

Simulation-based approaches to disease progression allow us to make counterfactual predictions about the effects of an untried series of treatment choices. However, building accurate simulators of disease progression is challenging, limiting the utility of these approaches for real world treatment planning. In this work, we present a novel simulation-based reinforcement learning approach that mixes between models and kernel-based approaches to make its forward predictions. On two real world tasks, managing sepsis and treating HIV, we demonstrate that our approach both learns state-of-the-art treatment policies and can make accurate forward predictions about the effects of treatments on unseen patients.

## 1 Introduction

Despite progress in machine learning methods for clinical decision support (e.g. [1–3]), machine learning algorithms usually operate as uninterpretable black-boxes which clinicians are often hesitant to trust and adopt as tools. Given this context, simulation-based approaches to managing disease progression are appealing because they allow us to make counterfactual predictions about the possible future outcomes associated with different treatment options. Especially in high-stakes decisions, simulatability can help guide and audit recommendations. For example, a clinician who sees that the current set of HIV treatments will lead to future drug resistance, may choose a different set of therapies. Alternatively, an intensivist may see a physiologically implausible blood-pressure trajectory accompanying a treatment recommendation and correctly decide to ignore the recommendation. In this way, simulations provide a complementary context than a set of guidelines or recommendations.

At its core, building a simulator requires building a model. In disease progression modelling, we commonly posit that a patient has some underlying (and unobserved) disease state *s* that evolves according to the choice of treatments or actions *a* they take, governed by some transition function *T*(*s*′|*s*, *a*). We assume that we cannot observe the true state of the patient, and can only measure partial observations *o*, governed by some probability function Ω(*o*|*s*, *a*). For example, in an oncology setting, the true disease state *s* might be patient’s cancer stage, while the observations *o* might be measured biomarkers and symptoms such as fatigue or weight loss. Given the model, we may subsequently use it to forward simulate potential histories and identify the most optimal treatments.

Unfortunately, disease progression is complex, and building models accurate enough for making decisions is challenging. Thus in many treatment recommendation settings, kernel-based regressors are much more common (e.g. [4, 5, 6]). These approaches work by identifying similar patients and recommending the (usually one-step ahead) action that worked best for those similar patients. Kernel-based regressors have also been built into models: [7, 8] and [9] all build dynamical system models that predict the patient’s next physiological state based on the next-states of the patient’s nearest neighbours. Using this kind of non-parametric predictor, rather than being confined to some parametric model, greatly improves model accuracy, especially if the underlying dynamics are complex and the data are dense.

However, kernel-based approaches to building models still have an important failure mode: because they work by matching patients with similar conditions, they perform poorly for patients with uncommon conditions. This limitation is an important concern for healthcare applications of kernel methods, as there often exists a large tail of distinct cases.

To address this challenge, we propose *kernelised dynamical mixing* (KDM), a hybrid approach that combines parametric (standard model-based) and non-parametric (kernel-based) predictors into one dynamical model of disease progression. Conceptually, when trying to predict how a specific patient’s disease will evolve given a specific intervention, we build a gating network that will select whether it is more accurate to use a kernel-based prediction, which can model more complex functions but extrapolates poorly, or a model-based prediction, which is simpler but therefore extrapolates more smoothly. We demonstrate that our approach allows us to make both better forward predictions of disease progression and better treatment recommendations than either alone. Specifically,

- We introduce a hybrid strategy called kernelised dynamic mixing (KDM) that permits dynamically combining parametric (model-based) and non-parametric (kernel-based) counterfactual predictions of events within a forward planning setting.
- On two real clinical tasks, managing HIV and managing sepsis, our KDM-based approach produces more accurate predictions of future disease states compared to either parametric or non-parametric models alone.
- On those tasks, we show our KDM-based approach not only makes better treatment recommendations than either parametric or non-parametric models alone, but also makes better treatment recommendations than other state-of-the-art, non-model-based approaches [4–6].

## 2 Related work

Kernel-based methods have a long history in reinforcement learning. Ormoneit and Sen [10] assess the value of a particular state by averaging over histories passing near it. Other works, notably [7, 8, 11, 12], use kernels to explicitly build models. For example, the authors of [7, 8] take a non-parametric view of learning policies by representing distributions over states, actions, and observations as embeddings in Hilbert spaces, and defining policies and value functions over these embeddings. Song et al. [11] establish a principled connection between Bayesian inference and posterior distribution embeddings via the kernel Bayes’ rule. Specifically, the authors express kernel Bayesian inference as a vector-valued regression problem and impose additional regularisation terms to control the resulting posterior embeddings, thus incorporating side information or domain knowledge into a problem. However, all of these approaches make predictions only from the data; while the choice of feature space may provide some regularisation effect, these approaches cannot be expected to generalise far from the observed histories.

Also related to our work, are methods that combine knowledge from different sources. The authors of [13–15] use rollouts with variants of experience replay to prevent sample degradation; they augment the training data used to learn a model with samples from a hallucinated context, and replay this experience to correct the model when it produces errors. Marco et al.[16] trade off knowledge from simulations and physical experiments by explicitly representing the costs of different sources of information in a Gaussian process model, and use an entropy-based search to minimise quality of information costs while optimising performance. Chebotar et al.[17] integrate model-based policy optimisation with model-free updates to improve a policy. While similar in spirit, Chebotar et al.[17]’s method is not designed to produce accurate future trajectories; it only aims to identify the optimal policy.

Other approaches try to capture model uncertainty more effectively. For example, [18, 19] use probabilistic transition models such as Gaussian processes to incorporate uncertainty in the transition distribution into planning. These approaches are best suited for continuous, low-dimensional action spaces—not the norm in healthcare applications—and neither combines models with data in forward planning as we propose here.

Finally, other works combine models and data at the *policy level*, rather than for forward simulation. Parbhoo et al. [20] recently proposed a Mixture-of-Experts (MoE) which switched between policies from a simple kernel regression and policies derived from a traditional state-space model learned on the same data. Applying this approach to produce HIV treatment recommendations, they found that for outlier patients, decisions based on a simplified model were better than incorrectly presuming treatment response would be similar to dissimilar patients. However, their approach cannot be used to *simulate* what might happen if the policy is followed. We instead propose an approach for combining kernel and model-based approaches on a *model* level.

## 3 Preliminaries and notation

Let $D={\left\{h_{nT_{n}}\right\}}_{n=1}^{N}$ be a collection of *N* patient histories of length *T*_*n*_ where each history is comprised of a sequence of treatments (actions) *a*, observations *o*, and outcomes (rewards) *r*, $h_{nT_{n}}=\left\{a_{n1},o_{n1},r_{n1},\ldots ,a_{nT_{n}},o_{nT_{n}},r_{nT_{n}}\right\}$. In general, the treatment that optimises a patient’s immediate outcomes do not necessarily guarantee a patient’s health in the long term. Our goal is to, for any patient history *h*, identify a policy *a* = *π*(*h*) or sequence of treatments that optimises a patient’s expected long-term outcomes $R:=E[\sum_{t=0}^{T}\gamma^{t}r_{t}]$, where *γ* is a discount factor that trades between the importance of current and future rewards.

Below, we describe three standard ways of deriving such a policy. The first two are model-based approaches: we first learn a parametric or non-parametric dynamical system model of disease progression, and then use that model to plan. The final approach is a non-parametric regression-based approach that directly learns the policy, without learning a model first.

### 3.1 Parametric models for dynamical systems

A common way to model decision-making processes such as therapy selection when a patient’s underlying state is unknown is via a partially observable Markov decision process (POMDP) [21]. A discrete-state POMDP *m* consists of a finite set of hidden states S, actions A, observations O; a transition function *T*(*s*′|*s*, *a*) that specifies the probability of transitioning from state *s* to *s*′ when taking an action *a*; an observation function Ω(*o*|*s*, *a*) that specifies the probability of observing *o* from state *s* when taking action *a*; and the reward function R:S×A→R determines the immediate reward *r* in state *s* when taking action *a*.

#### Summarising the history

In general, making decisions in a partially-observable setting requires the entire history. Fortunately, there exists a succinct sufficient statistic for the history: the belief *b* ≡ *p*(*s*|*h*), the distribution over states given the history. Given the belief *b*_*t*−1_, an action *a*_*t*_, and a new observation *o*_*t*_, the belief *b*_*t*_ can be computed via Bayes’ rule:

$$\begin{array}{c} b_{t}\left(s\right)=\Omega \left(o_{t}|s,a_{t}\right)\sum_{s^{\prime }\in S}\frac{T\left(s|s^{\prime },a_{t}\right)b_{t-1}\left(s^{\prime }\right)}{p(o_{t}|b_{t-1},a_{t})}, \end{array}$$ (1)

where $p\left(o_{t}|b_{t-1},a_{t}\right)=\sum_{s^{\prime }\in S}\Omega \left(o_{t}|s^{\prime },a_{t}\right)\sum_{s\in S}T\left(s^{\prime }|s,a_{t}\right)b_{t-1}\left(s\right)$.

#### Learning a policy

Model-based RL methods interleave between two phases: using available histories to estimate the transition function *T*(*s*′|*s*, *a*) and observation function Ω(*o*|*s*, *a*) [22], and using the learned model to derive a policy *π*(*b*, *a*) to maximise the long-term return $R=E[\sum_{t}\gamma^{t}r_{t}]$. In this work, we focus on online POMDP planners [23, 24] to derive the policy because they only require the ability to simulate from the model—something that both the parametric model above and the non-parametric model below will be able to provide.

Online POMDP planners operate by rooting a tree at the current belief *b*_*t*_. Next, the tree branches on each action *a* the agent may take and observation *o* the agent might observe. At each action node, the agent computes its expected immediate reward $R\left(a\right)=E_{s}\left[R\left(\cdot |s,a\right)\right]$. The value of taking action *a* in belief state *b*(*s*) is

$$\begin{array}{c} Q\left(a,b\right)=R\left(a,b\right)+\gamma \sum_{o}\Omega \left(o|b,a\right)\max_{a^{\prime }}Q\left(a^{\prime },b^{ao}\right), \end{array}$$ (2)

where *b*^*ao*^ is the agent’s belief after taking action *a* and observing *o* from belief state *b*, and *R*(*a*, *b*) = ∑_*s*_ *b*(*s*)*R*(*s*, *a*), and the action-value *Q*(*a*′, *b*^*ao*^) is recursively calculated down the tree to some depth *D*. Especially when the observation space is large, it is common to approximate the sum above with samples from Ω(*o*|*b*, *a*). Since the belief state *b* captures the entire history *h* of a patient, we refer to the term Ω(*o*|*b*, *a*) as Ω(*o*|*h*) for the rest of this paper. Thus, to perform this forward planning, we only require (a) a method to sample observations given the history and (b) a method to approximate the sufficient statistic for the history *b*.

### 3.2 Non-parametric models for dynamical systems

Dynamical systems may also be modelled non-parametrically for instance, in a kernel-based setting. Notable works that take this approach include [7], [11] and [8]. These approaches construct models specifically by representing distributions *T*(*s*′|*s*, *a*), Ω(*o*|*s*, *a*) and beliefs *b* as embeddings in Reproducing Kernel Hilbert Space (RKHS), and performing belief updates in accordance to Kernel Bayes’ rule [25]. Approaches based on Kernel Bayes’ rule can however be difficult to use in practice, as they require explicit knowledge about the hidden state in order to learn the embeddings of the distributions from training samples.

As an alternative to the aforementioned approaches, kernel-based learning may be used to directly sample observations *o*_*t*+1_. In this case, *o*_*t*+1_ may be drawn by considering the observations of the nearest neighbours and weighting these according to kernel function *k*(*h*_*t*_, ⋅). In doing so, it is possible to deduce a kernel-based probability estimate of $\Omega \left(o|h\right)\propto \sum_{h_{t}^{\prime }}k\left(h_{t},h_{t}^{\prime }\right)\delta \left(o=o_{t+1}|h_{t}^{\prime }\right)$ from which *o*_*t*+1_ may be sampled. Since the forward search in Eq 2 only requires simulations of the next observation, these observations may be incorporated directly into model-based planning. We build on this idea in this paper.

### 3.3 Kernel-based regression for direct policy learning

An alternative view poses the task of therapy selection as a data-driven non-parametric, regression problem, without learning a model first. Suppose we are given a set of pairs of patient histories and long-term return {*h*_*nt*_, *R*_*n*_}. For each history *h*_*nt*_, we can predict its long-term return $\hat{R}$ via a non-parametric regression where our predictions are expressed by averaging over nearby histories $h_{nt}^{\prime }$ as follows,

$$\begin{array}{c} {\hat{R}}^{\prime }=\sum_{h_{nt}^{\prime }}k\left(h_{nt},h_{nt}^{\prime }\right)R_{n},\forall h_{nt}^{\prime }\in H. \end{array}$$ (3)

Here, $k(h_{nt},h_{nt}^{\prime })\geq 0$ is a weighting kernel function in RKHS satisfying $\sum_{h_{nt}^{\prime }}k\left(h_{nt},h_{nt}^{\prime }\right)=1$, $\forall h_{nt}\in H$, and H represents the set of patient histories. Intuitively, this implies that one can assess the long-term value of taking an action *a* by examining the training data of histories where *a* has been applied and averaging over their long-term values; thus, at each time step, one can choose the action *a* that is predicted to maximize the long-term return without learning a model first. While necessarily approximate—this approach assumes one will follow the entire history of future actions, not just the next action, it often works well in practice.

## 4 Kernelised dynamic mixing

**Fig 1:**
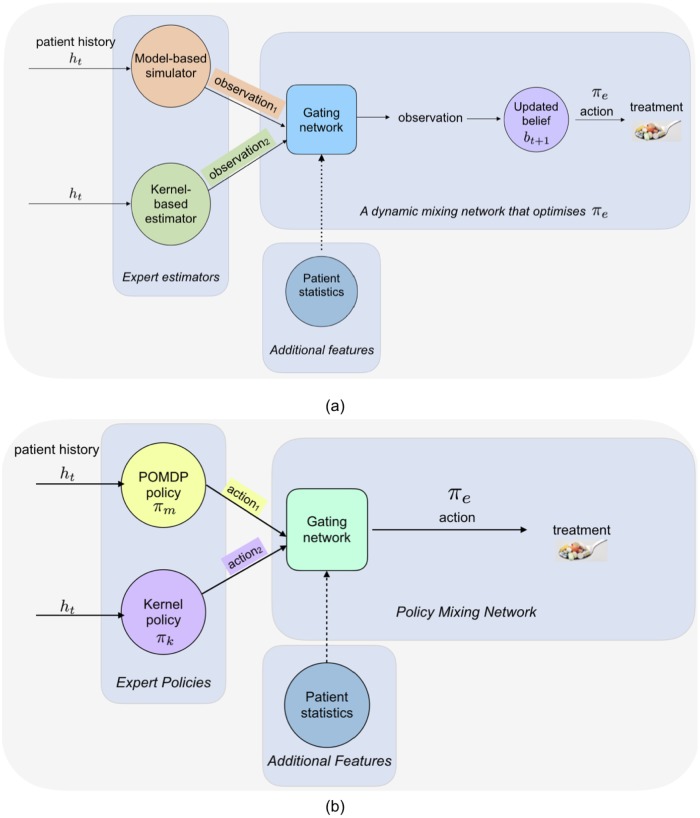
In our model-mixing approach (a), we create a simulator that chooses between parametric (discrete POMDP) and non-parametric (kernel) approaches for performing the forward simulation and use this simulator for planning. In contrast, earlier work (b) solved for a policy using either the POMDP or the kernel, and then chose between policies at test time. Given that both the POMDP and the kernel both have their respective weaknesses, we expect policies derived from just one to be less optimal than those derived from a model that can dynamically mix between both.

Both the parametric POMDP-based modelling approach in Section 3.1 and the non-parametric kernel-based modelling approach in Section 3.2 have their advantages: the simpler discrete POMDP tends to extrapolate better, whereas the kernel-based approach tends to be more accurate in regions of dense data. In this section, we present a modelling approach that dynamically mixes between these two approaches to build a simulator that is more accurate than either alone; given this simulator, we can then identify treatments using the online planner from Section 3.1. Importantly, because predictions are combined in an *model-based setting*, all the advantages associated with model-based approaches apply here. Through forward simulation, we can assess a treatment policy holistically in terms of the particular observations that may result from a particular choice of drug, and perform counterfactual reasoning about the subsequent series of events that may follow. We present an overview of our model-based approach in Fig 1, in contrast with policy-based mixing approach of [20] of earlier work.

### Main algorithm

Both the discrete POMDP and the kernel-based model can be used to sample future observations given a history. Our approach combines these predictions to make this simulation more accurate. Specifically, we consider models such that the probability of an observation given a history Ω(*o*|*h*) is a linear combination of the probabilities under the POMDP model Ω_*m*_(*o*|*h*) and the kernel-based approach Ω_*k*_(*o*|*h*):

$$\begin{array}{c} \Omega \left(o|h\right)=\theta \left(h\right)\Omega_{m}\left(o|h\right)+\left(1-\theta \left(h\right)\right)\Omega_{k}\left(o|h\right) \end{array}$$ (4)

where *θ*(*h*) ∈ [0, 1] is some mixing parameter that trades between the two estimates. (We do not consider learning transition and observation models directly because, as noted in [7], these would require access to the hidden state *s*.) We note that the mixing in Eq 4 is complementary to kernelised reinforcement learning approaches such as kernelised POMDPs and PSRs [7, 11]. Both of these approaches regularise the kernel-based predictions through a bottleneck of the belief over states or core test predictions. In contrast, we include the parametric POMDP model over future observations, Ω_*m*_, as an equal player in the prediction task, as if it were another special kind of patient history with kernel weight *θ*(*h*).

Once we have the function Ω(*o*|*h*), we can extend a history *h* given an action *a* by sampling from Ω(*o*|*h*). We can continue this forward simulation process for as long as we want; at each stage, we shall have a new history *h*′ to compare to the batch of our histories in the kernel-based model and a new belief *b*′ to be the sufficient statistic for our POMDP-based model. The final step to use this new simulator to optimise for new policies is to define the reward function on the basis of history *h*′. In our work, we use the POMDP alone to determine the immediate reward, although in principle the kernel could also be used. Our approach to using the POMDP to determine rewards is analogous to the approach from in [7]. Given the rewards we can now apply forward search to find an optimal policy via Section 3.1 (see description in Algorithm 1).

**Algorithm 1** Kernelised Dynamic Mixing Planner

**Require**:

 Θ(⋅, *W*): MLP prediction function, with parameters *W*

 $B={\left\{b_{t}\right\}}_{n=1}^{N}$: belief states for each patient at time *t*

 $H={\left\{h_{t}\right\}}_{n=1}^{N}$: histories of each patient at time *t*

 *k*(⋅, ⋅), Ω_*k*_: kernel parameters

 Ω_*m*_, *T*, *R*: POMDP parameters

1: **function** KDM(*θ*)

2:  **while** search depth has not been reached **do**

3:   Branch on an action *a*_*t*_

4:   Predict *θ* = Θ(⋅, *W*) based on *T*, *k*(⋅, ⋅), and history length

5:   Set Ω = *θ*(*h*_*t*_)Ω_*m*_ + (1 − *θ*(*h*_*t*_))Ω_*k*_

6:   Sample new observation *o*_*t*_ from Ω

7:   Use *o*_*t*_, *h*_*t*_ and *a*_*t*_ to predict *R*

8:   Update belief *b*_*t*_ according to *o*_*t*_ and *a*_*t*_ using Eq (1)

9:   Add *o*_*t*_, *a*_*t*_ and *r*_*t*_ to existing history *h*_*t*_

10:  Backpropagate values up through the search tree to get $a_{t}^{*}$

11:  **return** Updated *b*_*t*_ and optimal action $a_{t}^{*}$

### Learning the mixing proportion *θ*(*h*)

The key question, of course, is how to define the mixing function *θ*(*h*) to make our probability of observation estimate Ω(*o*|*h*) in Eq 4 as accurately as possible for new histories. To do so, we note that while at test time the next observation *o*_*t*+1_ is not observed, our training set will contain many histories that can be cut into some past history and some next observation. That is, we have access to *o*_*t*+1_. Thus we can consider

$$\begin{array}{c} \max_{\theta }\frac{1}{N}\sum_{n}^{N}\frac{1}{T_{n}}\sum_{t}^{T_{n}}\text{log}\left(\theta_{nt+1}\Omega_{m}\left(o_{t+1}|h_{nt}\right)+\left(1-\theta_{nt+1}\right)\Omega_{k}\left(o_{t+1}|h_{nt}\right)\right) \end{array}$$ (5)

In the formulation above where our goal is to predict the true next observation correctly, we note that either the POMDP or the kernel must necessarily be more accurate; thus, the optimal choice of *θ*_*nt*_ at any time will be to select that more accurate model. During training, rather than fit to a binary target, we consider the softmax version

$$\begin{array}{c} \theta \left(h_{nt}\right)≔\frac{\text{exp}(\Omega_{m}\left(o_{t+1}|h_{nt}\right))}{\text{exp}\left(\Omega_{m}\left(o_{t+1}|h_{nt}\right)\right)+\text{exp}(\Omega_{k}\left(o_{t+1}|h_{nt}\right))}. \end{array}$$ (6)

The softmax target is akin to having a classifier probabilistically predict which method makes most sense to use at each point in time. Specifically, it provides a probabilistic interpretation of which method is more likely to produce the observed future values, and hence determines which method should be given a higher weight for that time step.

Finally, we note that while one could train the weighting term *θ* to simply be a function of the history *h*, that is, some *θ*(*h*), the *relationship* between the history of interest *h* and the other histories in the training set is very important—as we mentioned before, we expect the kernel-based approach to be more accurate in regions where the data are dense and the POMDP to be more accurate otherwise. Thus, we include additional inputs to the predictor *θ*: patient statistics in terms of the history length of the current history *h*, along with the 5-quantiles of the function *k*(*h*, ⋅) with respect to the training set. We call this collection of statistics *ς*, so our predictor is now *θ*(*ς*).

Given the batch of histories, we can now create a collection {*ς_nt_*, *θ_nt_*}, where *ς_nt_* are the properties of the history and its relationship to the data and *θ*_*nt*_ is the softmax target (Eq 6). We train a multilayer perceptron (MLP) Θ as a mixing network to predict *θ*_*nt*_ given parameters *ς*. Let vector *W* denote the parameters of the MLP. Then we write the training objective as

$$\begin{array}{c} \min_{W}\sum_{n,t}{\left(\theta_{nt}-\Theta \left(\varsigma_{nt},W\right)\right)}^{2}+{\lambda ||W||}_{2}^{2}, \end{array}$$ (7)

This loss is differentiable, and thus we can optimise it with gradient descent.

## 5 Experiment setup: Evaluation measures and baselines

Our experiments focus on two related goals: (1) to characterise the performance of KDM in comparison in existing baselines, and (2) to assess the quality (in terms of forward predictions) and interpretability of approach in comparison to existing methods. Below we describe our metrics as well as our baselines.

### 5.1 Evaluation: Forward simulation quality

The KDM procedure described in the previous section provides a principled means of dynamically integrating kernel-based predictions into model-based RL to not only learn suitable treatment policies, but also provide counterfactual predictions. It is relatively straightforward to evaluate the quality of the predictions on retrospective data—at any time point, we have our distribution over possible next-observations, and we can compute the log-loss with respect to that distribution given what observation actually occurred. Additionally, we provide illustrations of the deviation between our counterfactual predictions and the ground truth in terms of the observations produced.

### 5.2 Evaluation: Policy quality

While evaluating the quality of the forward simulation (above) was relatively straight-forward, evaluating policy quality is much more difficult. We apply a collection of importance-sampling based estimators to evaluate our policies. (We report several, because each have different bias-variance trade-offs.) Conceptually, all of these methods try to determine a subset of the data over which the behavioural policy, *π*_*b*_, coincides with the evaluation policy *π*_*e*_.

The classic IS estimator [26–28] over the the value function *V* is given by,

$$\begin{array}{c} V^{\pi_{e}}=\sum_{n=1}^{N}w^{h_{n}}R^{h_{n}}, \end{array}$$ (8)

where *h*_*n*_ is the history of a patient *n* of length *T*_*n*_, $R^{h_{n}}$ is the total reward accumulated over the patient’s history, and $w^{h_{n}}$ is an importance ratio of that reflects how likely a history *h*_*n*_ is under the evaluation policy. Here, histories that are unlikely are given a smaller weight when evaluating a policy. The importance ratios $w^{h_{n}}$ may be computed according to,

$$\begin{array}{c} w^{h_{n}}=\prod_{t=0}^{T_{n}}\frac{\pi_{e}\left(a_{t}^{h_{n}}|b_{t}^{h_{n}}\right)}{\pi_{b}\left(a_{t}^{h_{n}}|b_{t}^{h_{n}}\right)} \end{array}$$ (9)

Since the IS estimator is unbiased but prone to high variance, a variant known as weighted-IS is often used for off-policy evaluation. This estimate can be computed as a weighted average of the samples,

$$\begin{array}{c} V^{\pi_{e}}=\frac{\sum_{n=1}^{N}w^{h_{n}}R^{h_{n}}}{\sum_{n=1}^{N}w^{h_{n}}}. \end{array}$$ (10)

While the estimate has a lower variance than IS, it is however biased. The doubly robust off policy evaluation scheme (DR) [29] attempts to address this trade-off between bias and variance by coupling the IS weights from Eq 9 with a regression estimate $\hat{Q}$ of the value function $V^{\pi_{e}}$ (computed on a separate data set). The estimated value of *π*_*e*_ can then be computed using,

$$\begin{array}{c} V^{\pi_{e}}=V^{\pi_{e}}+\sum_{n=1}^{N}w^{h_{n}}\left(R^{h_{n}}-\hat{Q}\right) \end{array}$$ (11)

This evaluation scheme works well if either $\hat{Q}$ or the IS weights are reasonably accurate. It is important consider that while each of these IS-based estimators has its advantages, all the IS-based estimators have the limitation of assuming that the belief as a sufficient statistic for the state.

### 5.5 Baselines

For each of our experiments, we compare the performance of a policy obtained from KDM to several baselines. Our first baseline is a policy based on a non-parametric (kernel-based) model as described in Section 3.2. The policy is computed by estimating the long-term reward from the samples falling in an *ϵ* radius of a particular patient at a certain time point. The kernel policy successively applies the action from the nearby samples associated with the largest expected long-term reward. Note that despite the similarities KDM shares with the Hilbert Space Embedding of the POMDP (kPOMDP) [7], we cannot directly compare them since the kPOMDP requires knowledge of the true state representation during training—a severe limitation of the approach that makes it largely infeasible in practice. Here, the non-parametric model is used to approximate the kPOMDP. We also compare the KDM policy against a policy computed using a POMDP model alone. The third baseline is a MoE as described in [20], where we combine both parametric and non-parametric policy estimates using a gating network and choose actions accordingly. Across all tasks, we make the simplifying assumption that the belief state is a sufficient statistic for the history, and thus the policy is a function of the belief *π*(*b*).

### 5.4 Training parameters

To optimize the loss in Eq (7) we use L2 regularisation with strength λ > 0 and perform cross-validation against the true values of *θ*. We use *J* = 500 labeled pairs for training the mixing network on a toy example and *J* = 4000 for real world datasets. Optimisation of the mixing network’s objective is done via gradient descent. We use Autograd [30] to compute gradients of the loss in Eq (7) with respect to *ξ*, then use Adam [31] to compute descent directions with step sizes set to 0.01 for the toy experiment and 0.001 for the medical applications. Across all three tasks a discount factor of *γ* = 0.9 is used, which puts weight on not only immediate rewards, but also long-term future rewards. In doing so, we can optimise not only a patient’s immediate, but also their long-term health outcomes. (We do not use a very large *γ* as the domain does not require a particularly deep look-ahead to solve.) Further details of the training parameters are discussed in the next section.

## 6 Results

Below we show results on three domains. The first is a synthetic domain that highlights the how mixing parametric and non-parametric approaches when building a model can be beneficial. Next, we present two medical applications for administering treatments for patients with HIV and sepsis. In both cases, we present a quantitative evaluation of the policy and the forward simulation (note that for the forward simulation, we can only compare the model-based approaches; the MoE cannot produce counterfactual predictions). Our KDM approach produces better policies and is able to simulate counterfactual scenarios more accurately than the baselines.

### 6.1 Demonstration on a synthetic domain

Consider a system that evolves deterministically through 4 states: *S*_1_, *S*_2_ or *S*_3_, and finally absorbs in *S*_4_. Each agent has a variant that belongs to one of two types: A and B. Agents with variants of type A deterministically go through state *S*_2_, and agents with variants of type *B* deterministically go through *S*_3_. At each stage, there are three actions available: 0, 1 or 2. At each time step, the agent observes its variant (which is one of the two types), as well as its reward, which is given by:

$$\begin{array}{llll} S_{1}\{\begin{array}{l} r(a_{0})=-10\\ r(a_{1})=5\\ r(a_{2})=5 \end{array} & S_{2}\{\begin{array}{l} r(a_{0})=0\\ r(a_{1})=5\\ r(a_{2})=-10 \end{array} & S_{3}\{\begin{array}{l} r(a_{0})=0\\ r(a_{1})=-10\\ r(a_{2})=5 \end{array} & S_{4}\{r=0. \end{array}$$

**Fig 2:**
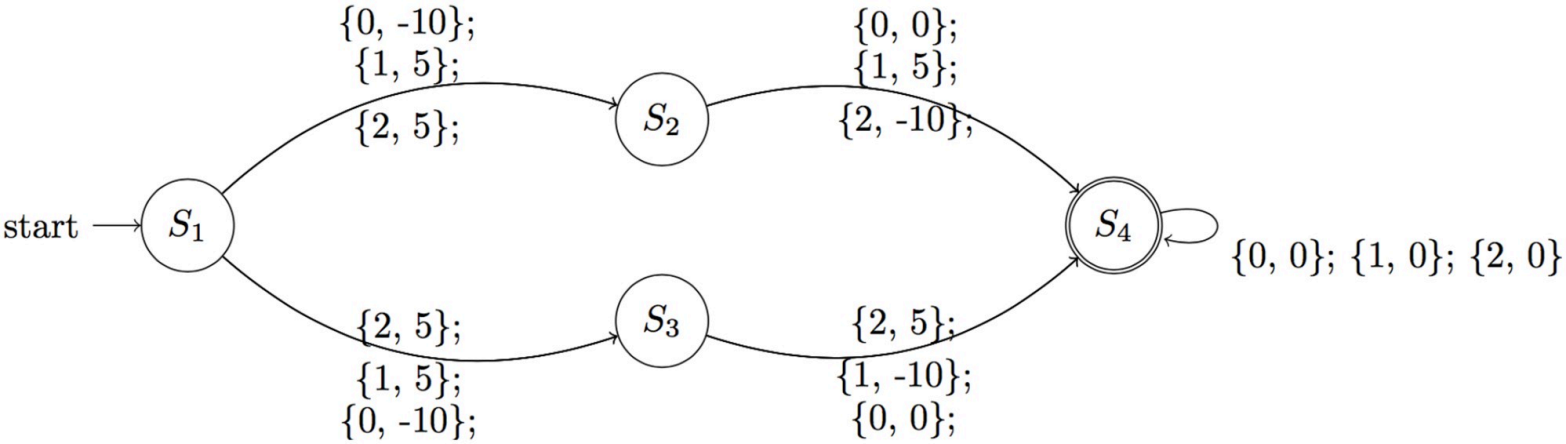
Illustration of dynamics for the toy example. The optimal sequence of actions for a type A variant is to initially take action 1 or 2, followed by action 1. For type B variants, the optimal sequence of actions is to first take actions 1 or 2, followed by action 2.

The optimal policy for all agents is to initially take either action 1 or 2. Next, agents with variants of type A transition to *S*_2_ where the optimal action is action 1; agents with variants of type B transition to *S*_3_ where the optimal action is action 2. Action 0 is safe in states *S*_2_ or *S*_3_. By construction, a four-state POMDP cannot learn the optimal policy for this model since the dynamics depend on the hidden type of the agent’s variant. Without the variant information, from the POMDP’s perspective, it is equally likely to transition from *S*_1_ or *S*_2_ starting from *S*_0_; not knowing where it will end up, it will initially suggest the safe policy of selection action 0 at the second time-step. For the kernelised planning approach, we use a kernel that matches based on the length of the agent’s history, action choices, and an observation dependent on the hidden variant. Such a choice will lead to optimal policies for agents with common variants. However, agents with rare variants will match to some arbitrary other agent, and we can expect the performance of the kernelised planner for those agents to be poor. In such cases, falling back on the POMDP will produce the optimal policy. An illustration of the toy example is shown in Fig 2. The numbers in brackets indicate the action taken from a particular state, followed by the associated reward.

We compared the performance of KDM against the baselines described earlier in this section, using a forward search depth of 4 (source code available at: https://github.com/dtak/dynamic-mixing). Our mixing network for KDM consists of 15 input units and a hidden layer of 25 units. We trained the models using a data set of *N* = 250 sequences, each with *T*_*n*_ = 4 time steps. A separate test set of the same size was used for evaluating performance. Table 1 compares the performance of KDM against the aforementioned baselines. The toy example illustrates that dynamically mixing kernel and model-based methods during simulation outperforms using either approach on its own. The quantitative differences between KDM and MoE policies suggest that combining parametric and non-parametric predictions on a model level results in different policies than combining these approaches on a policy level. Specifically, on a test set of 250 sequences, KDM learns the optimal policy 92% of the time, while in comparison the MoE approach learns the optimal policy 87% of the time.

**Table 1:** Performance comparison of KDM vs. baselines across 250 test sequences for the toy example. A higher value corresponds to a higher accumulated reward, and indicates a better performing policy.

	DR	WIS	IS
Random	–5.84 ± 2.61	–7.79 ± 3.71	–8.46 ± 3.24
Kernel	4.39 ± 1.74	4.86 ± 2.85	4.14 ± 2.72
POMDP	3.09 ± 1.16	3.62 ± 1.71	3.84 ± 2.42
MoE	5.62 ± 1.02	5.81 ± 2.37	5.42 ± 2.74
**KDM**	**6.08 ± 1.14**	**6.19 ± 1.03**	**6.32 ± 1.46**

### 6.2 HIV therapy selection

#### Cohort

Data for these patients were obtained from the EuResist database [32]. We extracted the genotype and treatment response data of *N* = 32960 patients together with their CD4^+^ and viral load measurements, gender, age, risk group and prior recorded treatments. The measurements are collected at approximately 6 month intervals corresponding to hospital visits. Variables with excessive missingness were removed, and any remaining missing values were imputed. We restrict the space of therapy combinations to the 312 most frequently occurring combinations in the cohort. These drug combinations span 20 drugs in total. Table 2 provides a summary of the cohort statistics used.

**Table 2:** Summary of HIV cohort statistics.

Number of Patients	32960
Average Sequence Length	14
Feature Dimensionality	134
Number of Actions	312

#### Reward function

Our goal in this task is to learn a policy that optimises a patient’s immune response to the virus, while simultaneously reducing the number of viral particles in the bloodstream. The immune response for HIV is frequently quantified in terms of CD4^+^ cells (e.g.[33]). To meet this goal, we propose the following short-term reward criterion:

$$r_{t}=\{\begin{array}{ll} -0.7\;\text{log}\;V_{t}+0.6\;\text{log}\;C_{t}-0.2|M_{t}|, & \text{if}\;V_{t}\;\text{is}\;\text{above}\;\text{detection}\\ 5+0.6\;\text{log}\;C_{t}-0.2|M_{t}|, & \text{if}\;V_{t}\;\text{is}\;\text{below}\;\text{detection}, \end{array}$$

where *V*_*t*_ is the viral load (in copies/mL), *C*_*t*_ is the CD4^+^ count (in cells/mL), and |*M*_*t*_| is the number of mutations at time *t* respectively. This function is identical to the reward function presented in [20] and is largely based on earlier work by [33]. It penalises instances where a patient’s viral load increases and rewards instances where a patient’s CD4^+^ count increases. It also penalises on the basis of the number of mutations a patient has at a particular time, as these may ultimately contribute to resistance and therapy failure. Like [20], we specifically place a higher weighting on the viral load than the CD4^+^ count or mutations, as it is often an earlier indication of treatment failure. Hence, a negative *r*_*t*_ corresponds to having a high viral load and potentially a large number of mutations. There is also a bonus for if the viral load is below detectable limits to sustain this over time. Summing *r*_*t*_ over a patient’s future allows us to explicitly quantify a patient’s response to therapy over this period. Specifically, a larger accumulated reward corresponds to having a small viral load and a strong immune response. In summing over *r*_*t*_, we can thus determine which policies are likely to improve a patient’s immune response in the long-term. While many alternative choices of reward function are possible here, HIV patient outcomes are typically quantified in terms of blood counts and viral load (See S1 Appendix for details). We also tested alternative choices of reward functions where we varied the weighting proportions of CD4^+^ and viral RNA. These results can be found in Tables A, B and C in S1 Appendix. Importantly, the dynamic mixing procedure presented in this paper is sufficiently general to be applied to any choice of reward function.

#### Experimental setup

We performed a random 80%-10%-10% train-test-validation split of our cohort of patients and compared the performance of KDM against the baselines. This split resulted in a held-out test set consisting of 3000 patients with the same distribution as patients in the training set. The training set was the largest split as we needed to learn the large number of parameters governing the kernel, POMDP, and dynamic mixing network.

The random policy selects a therapy randomly for each forward time step across all patients. For the kernel policy, we use the alignment kernel based on [4]. This kernel compares therapy histories of patients on the basis of the drugs that are used and the order in which they are administered, as well as in terms of the subsequent mutations that they produce. Two therapy histories are considered similar if they contain similar drugs, which are administered in a similar order, and produce similar mutations. For the POMDP policy, we learn a POMDP model with 30 states with Gaussian emissions, and observation spaces comprised of the demographic data, viral loads, CD4^+^ counts and genetic mutations that may occur as a result taking therapy. The number of states for the POMDP model is selected according to the Bayesian Information Criterion (BIC).

For planning, we perform a forward search for therapy choices that optimise patient outcomes over a 30-month horizon (corresponding to 5 forward time steps, which was chosen for tractable planning). Our mixing network for KDM consists of 100 input units and 2 hidden layers of 50 units each, where the number of parameters is selected by performing cross-validation on an independent hold-out set. Since the problem is non-linear by nature, our mixing network requires enough parameters to adequately approximate a smooth mapping between inputs and the mixing proportion. At the same time, over-parameterisation results in overfitting. To prevent the latter, we use regularise the network with an L2 regularisation of strength λ = 15.

#### Results

Table 3 summarises the performance of KDM compared to the aforementioned baselines. The KDM policy produces the highest accumulated immune response while reducing the viral load, outperforming the other baselines over a 30-month long-term horizon. The choice of time horizon is made on the basis of how frequently an HIV patient visits the hospital for treatment, medical guidelines and drugs available. Patient visits usually occur on a bi-annual basis, while medical guidelines and available drugs for treating HIV may change over longer periods of time. In general however, KDM may also be applied to extended time horizons.

**Table 3:** Performance comparison of KDM vs. baselines for HIV therapy selection across 3000 held-out patients using a POMDP model with 30 states. KDM produces the largest immune response while reducing the viral load, thus outperforming its competitors.

	DR	WIS	IS
Random	–7.31 ± 3.72	–11.48 ± 4.36	–10.64 ± 4.81
Kernel	9.35 ± 2.61	6.42 ± 3.93	6.73 ± 3.62
POMDP	3.37 ± 2.15	3.86 ± 2.38	3.74 ± 2.46
MoE	11.52 ± 1.31	12.25 ± 2.01	11.36 ± 2.97
**KDM**	**12.47 ± 1.38**	**14.25 ± 1.27**	**14.48 ± 1.41**

From observing the quantitative differences between the performance of KDM and the MoE policy, we can conclude that both the policies are different. Importantly, the model-based nature of KDM has several key benefits (particularly in a high-risk setting such as therapy selection). We highlight these differences with a motivating example: Consider an HIV-infected patient whose underlying health status is unknown, but with a baseline viral load of 589 copies/mL. If a patient is treated with a first-line therapy of EFV/3TC/TDF, we obtain a set of observations and rewards from which subsequent treatments may be selected. Based on the treatment of EFV + 3TC + TDF and the patient’s particular observations, KDM predicts that the viral load will drop below detection limits for a period of 6 months (which may or may not change the patient’s overall health status). At 12 months, KDM predicts that the virus reappears in the patient’s bloodstream, but falls below detection limits again shortly after this period. The MoE policy suggests a treatment change at 12 months from first line therapy to a more aggressive second-line therapy of AZT + 3TC + TDF + LPV/r.

**Fig 3:**
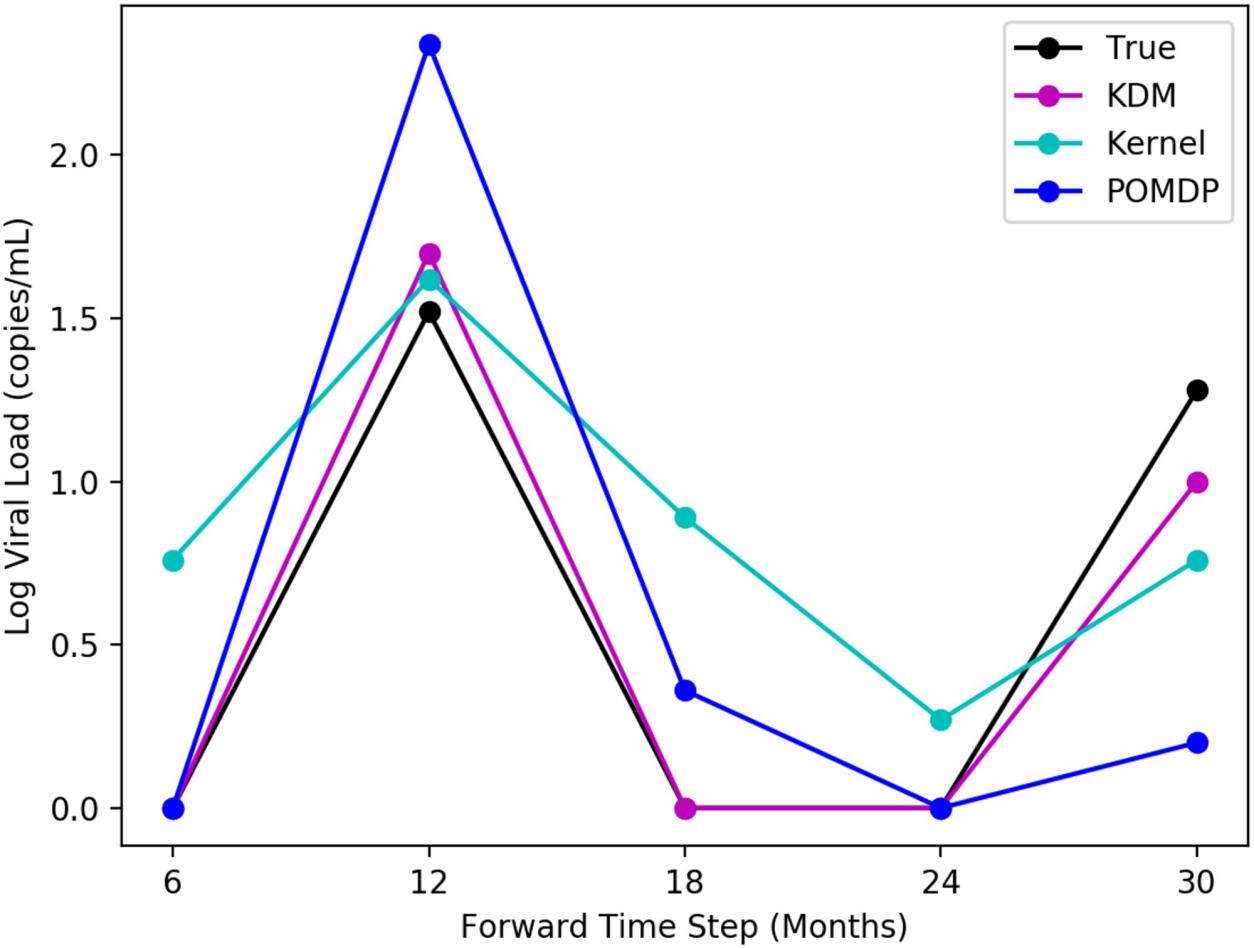
Simulating the viral load in an HIV patient when the viral load is below detection limits (indicated by 0). KDM can detect the occurrence of blips at 12 and 30 months, unlike a MoE. No treatment change should be administered here.

**Fig 4:**
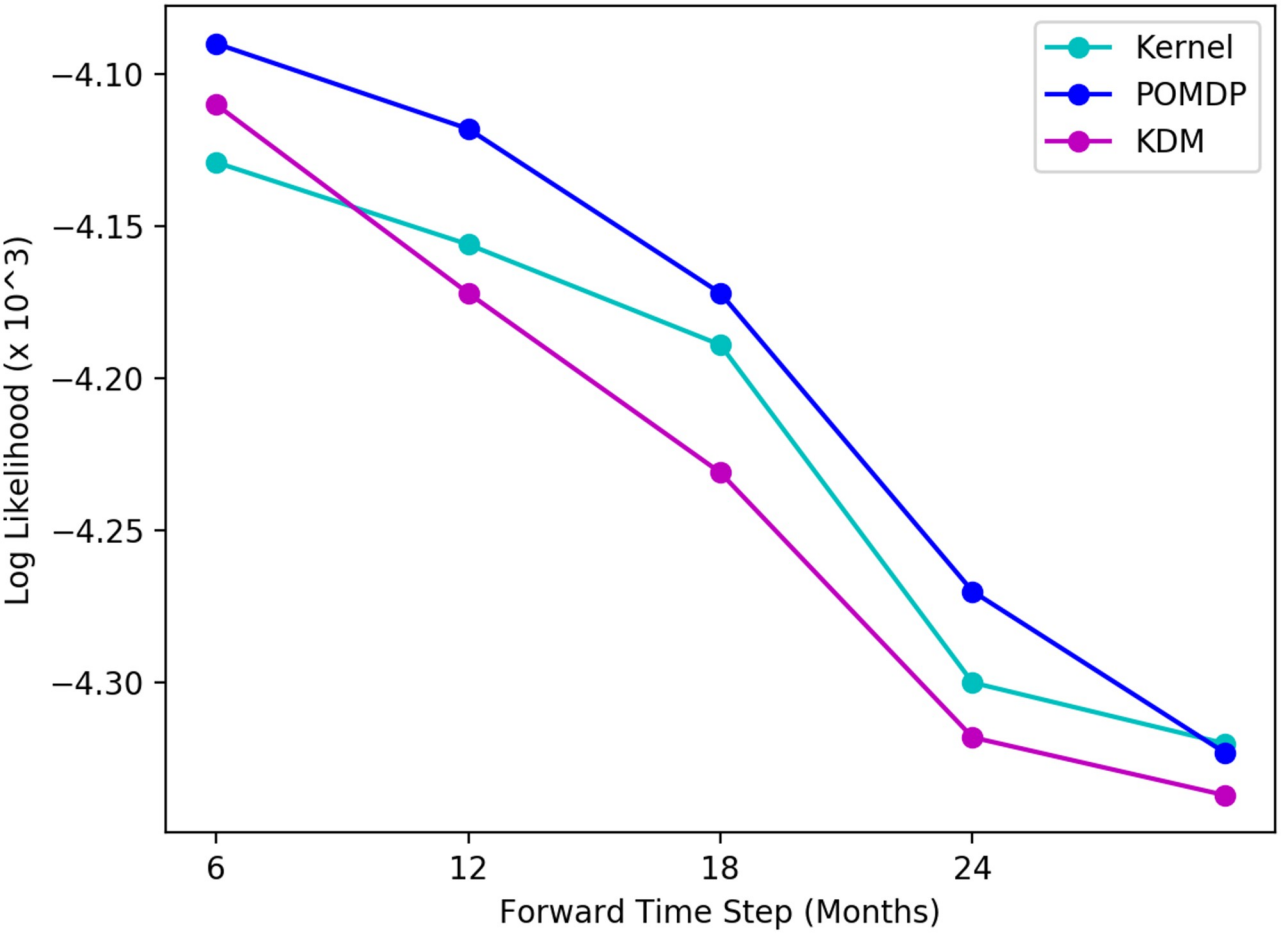
Comparison of predictive log-likelihood across baselines for HIV for a typical test patient. KDM’s predictions are more accurate across the forward time steps.

Because however, KDM actively simulates a patient’s future trajectory, it is able to predict the occurrence of a blip in the viral load at 12 months. As a result, the KDM policy continues using the same first-line therapy over this period, without suggesting a change in treatments. The implications of this are important: through actively forward simulating a patient’s long-term future, we can analyse the impact of making treatment decisions in terms of the particular outcomes that they may produce. The example here, highlights the fact that KDM is able to forward simulate occurrences of blips in the viral load and use this information to deduce whether or not a switch is necessary. In this case, switching treatments to a more aggressive treatment is unnecessary and potentially reduces a patient’s future therapy options. Importantly, the KDM policy may be easily interpreted through explicitly examining and auditing our forward simulations. This interpretability is key to building trust in machine learning methods in high-risk settings. Fig 3 illustrates forward simulating the viral load for the test patient described here. The ground truth, and respective kernel and POMDP-based predictions are shown. Since the MoE approach combines kernel and model-based learning on a *policy* level, it is impossible to obtain a set of forward predictions of a patient’s viral load (hence we cannot illustrate a trajectory for it here). The corresponding predictive log-likelihood is shown in Fig 4. Here, KDM’s forward predictions are closer to the ground truth and ultimately result in learning a more effective treatment policy overall. While obviously a single-patient anecdote, we found many such situations in which the KDM predicted deviations in trajectories.

**Fig 5:**
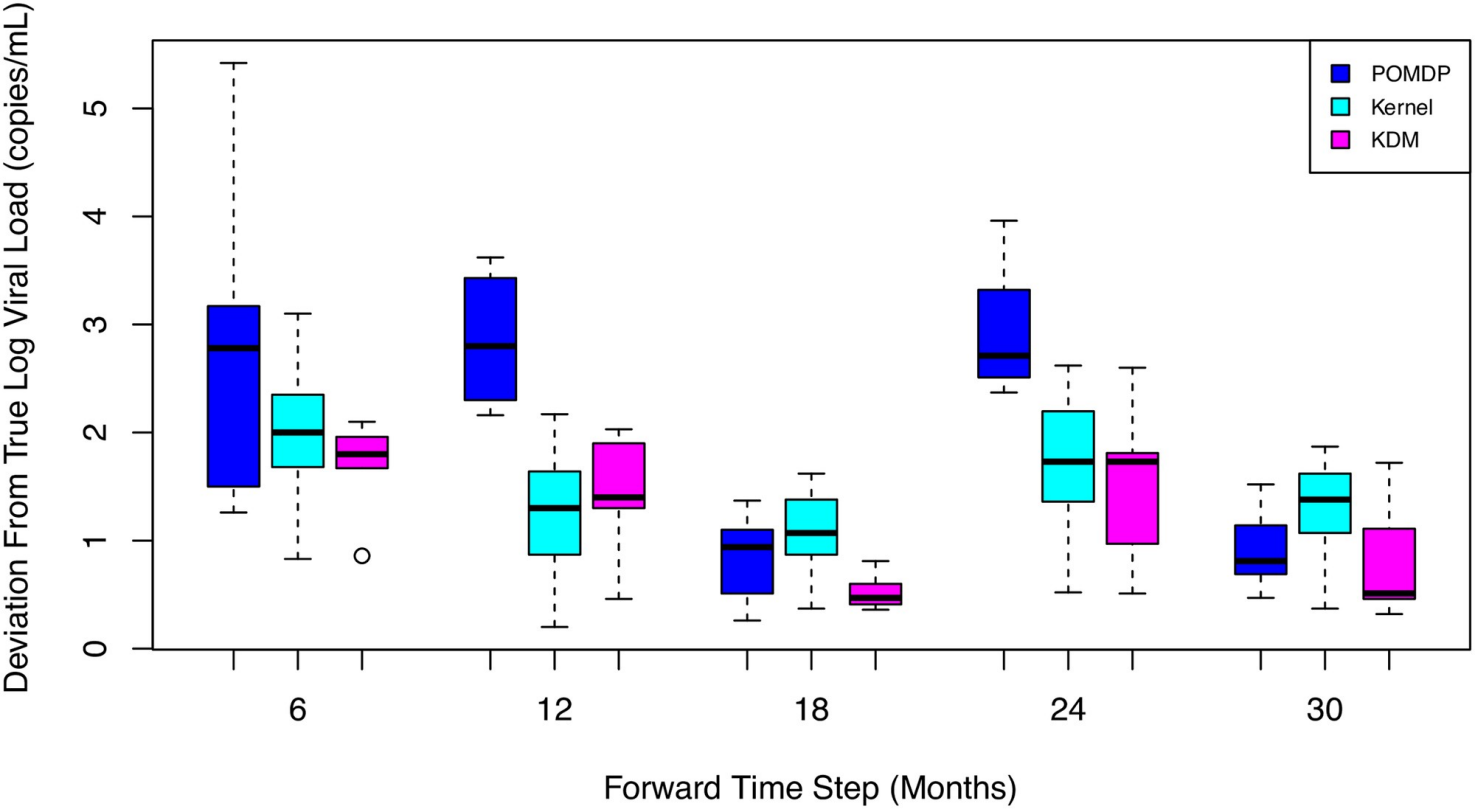
Box plot of viral load predictions across 3000 test patients under baselines over a 30-month horizon. KDM’s predictions are closer to the ground truth than POMDP or kernel predictions.

We obtain similar results on the rest of the patients in the test set. Fig 5 illustrates the deviations in counterfactual predictions of the viral load over a 30-month horizon. KDM is able to model and predict counterfactuals more accurately than the other baselines. This performance is sustained across all time steps.

### 6.3 Sepsis management

#### Cohort

Data for these patients were obtained from the publicly available Multiparameter Intelligent Monitoring in Intensive Care (MIMC-III v1.4) database [34], containing hospital admissions for approximately 38600 adults (at least 15 years old). We extracted a cohort of patients fulfilling Sepsis-3 criteria [35]. A summary of the populations can be found in Table 4. We extracted the appropriate physiological parameters such as demographics, lab values, vital signs and intake-output events. The data were aggregated into 4 hour windows, where the mean or sum was recorded (as appropriate) when several data points were present in a window. Variables with excessive missingness were excluded, and other missing values were imputed. This produced a feature vector of size 47 × 1 per patient for each time step. The values of each feature were passed through a sigmoid function to reduce the effect of outliers and subsequently normalised to zero mean and unit variance.

**Table 4:** Summary of sepsis cohort statistics.

Number of Patients	18200
Average Sequence Length	13
Feature Dimensionality	47
Number of Actions	100

The action space of medical interventions was defined to cover the space of intravenous (IV) fluid, and maximum vasopressor (VP) dosage, as well as whether or not to sedate and ventilate a patient in a given four hour window. We discretised the action space into per-drug quartiles based on all non-zero dosages of the two drugs, and converted each drug at every time step into integer values representing the respective quartile bin. We included a special case of no drug given as bin 0. This created an action representation of interventions as tuples of (total IV in, maximum VP in, sedation, mechanical ventilation) at each time step.

#### Reward function

Our overall goal in this task is to reduce patient mortality. Mortality, however, is a sparse outcome: whether a patient survived can only be known at the end of the stay. At the recommendation of our clinical colleagues, we use the log odds of in-hospital mortality as described in [36, 37] as an intermediate cost function for treating sepsis at each time step (we note, more broadly, that there exists relatively little clinical literature on optimisation criteria for sepsis). This reward function is trained on a held-out subset of the sepsis data cohort. Summing the log odds of in-hospital mortality over a patient’s future allows us to explicitly quantify a patient’s odds of mortality over this period. Since our goal is to reduce mortality, a lower accumulated cost corresponds to a better performing treatment policy in this case. (We also emphasise that our dynamic mixing procedure is general in that it can be applied to any cost or reward function, and retrained as domain experts refine their cost functions.)

#### Experimental setup

Once again we performed a random 80%-10%-10% train-test-validation split of our cohort of patients and compared the performance of KDM against the baselines on a held-out set of 3000 patients. For the kernel policy, we use a kernel that matches based on the length of the agent’s history, action choices, and observations. For the POMDP policy, we learn a POMDP model with 75 states with Gaussian emissions, corresponding to the observation space of lab values, vital signs and intake-output events described above. Once again, the number of states for the POMDP model is selected according to the BIC.

For the planning, we perform a forward search for therapy choices that optimise patient outcomes over a 20-hour horizon, again corresponding to 5 forward time steps that was both the limit of tractable planning and reasonable given that stays in the ICU are relatively short. Our mixing network for KDM consists of 40 input units and 2 hidden layers of 25 units each. The number of network parameters is again selected by performing cross-validation on an independent hold-out set.

#### Results

Table 5 summarises the performance of KDM compared to the aforementioned baselines for sepsis management. The KDM policy significantly reduces the risk of mortality for held-out patients over a 20-hour horizon, once again outperforming the other baselines.

**Table 5:** Performance comparison of KDM vs. baselines for treating sepsis across 3000 held-out patients using a POMDP model with 75 states. The KDM policy significantly reduces the odds of mortality (indicated by a lower value here), and outperforms existing baselines.

	DR	WIS	IS
Random	4.31 ± 1.72	3.52 ± 1.76	4.26 ± 1.82
Kernel	-0.88 ± 0.41	-1.47 ± 0.33	-1.63 ± 0.48
POMDP	1.73 ± 1.69	1.73 ± 1.25	1.86 ± 1.29
MoE	-1.42 ± 0.71	-1.85 ± 0.57	-1.46 ± 0.79
**KDM**	**-1.87 ± 0.39**	**-2.25 ± 0.77**	**-2.86 ± 0.80**

**Fig 6:**
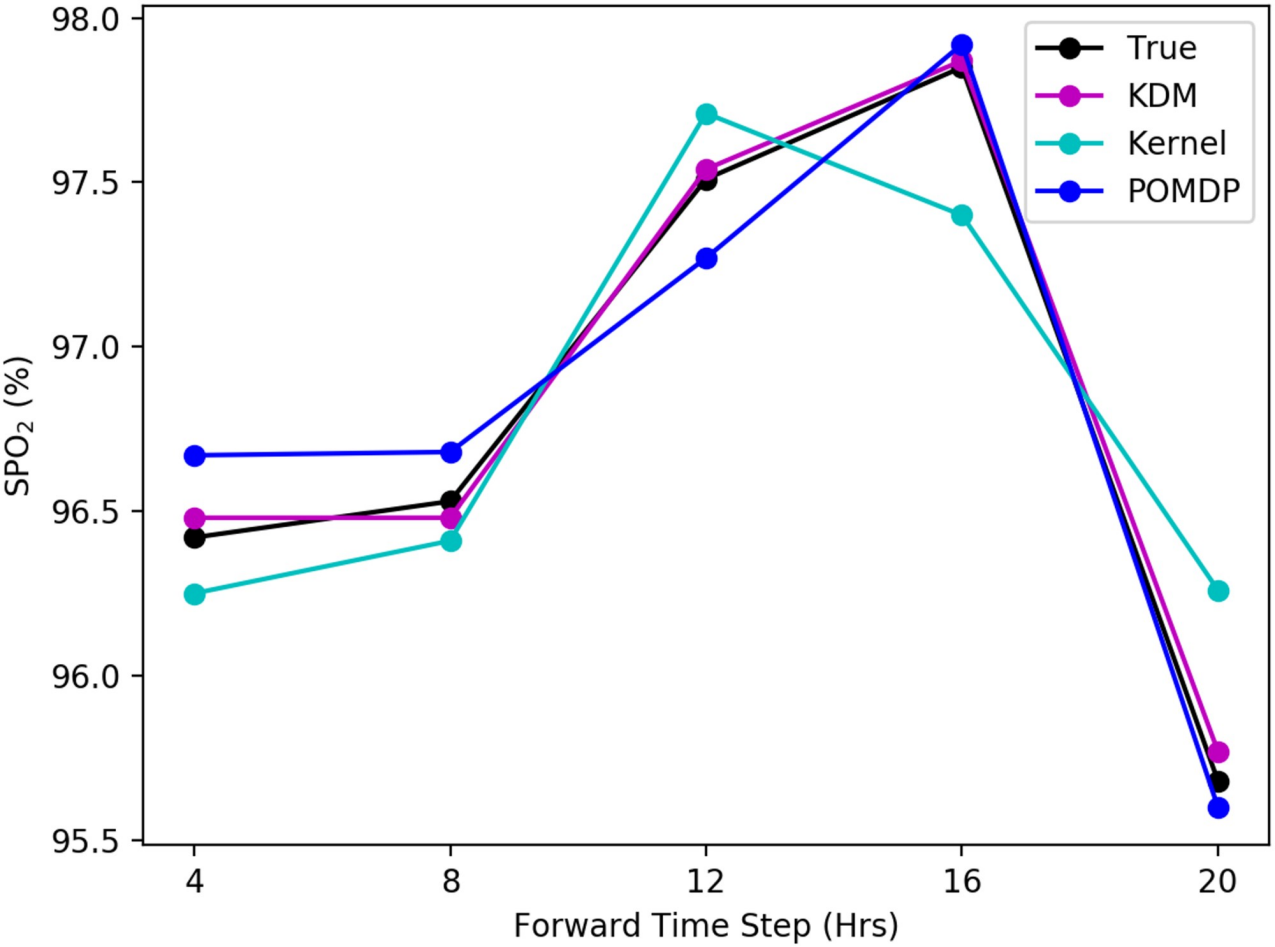
Simulating the SpO_2_ of a sepsis test patient under baselines over a 20-hour horizon. Counterfactual predictions of SpO_2_ levels are more accurate using KDM than existing baselines.

**Fig 7:**
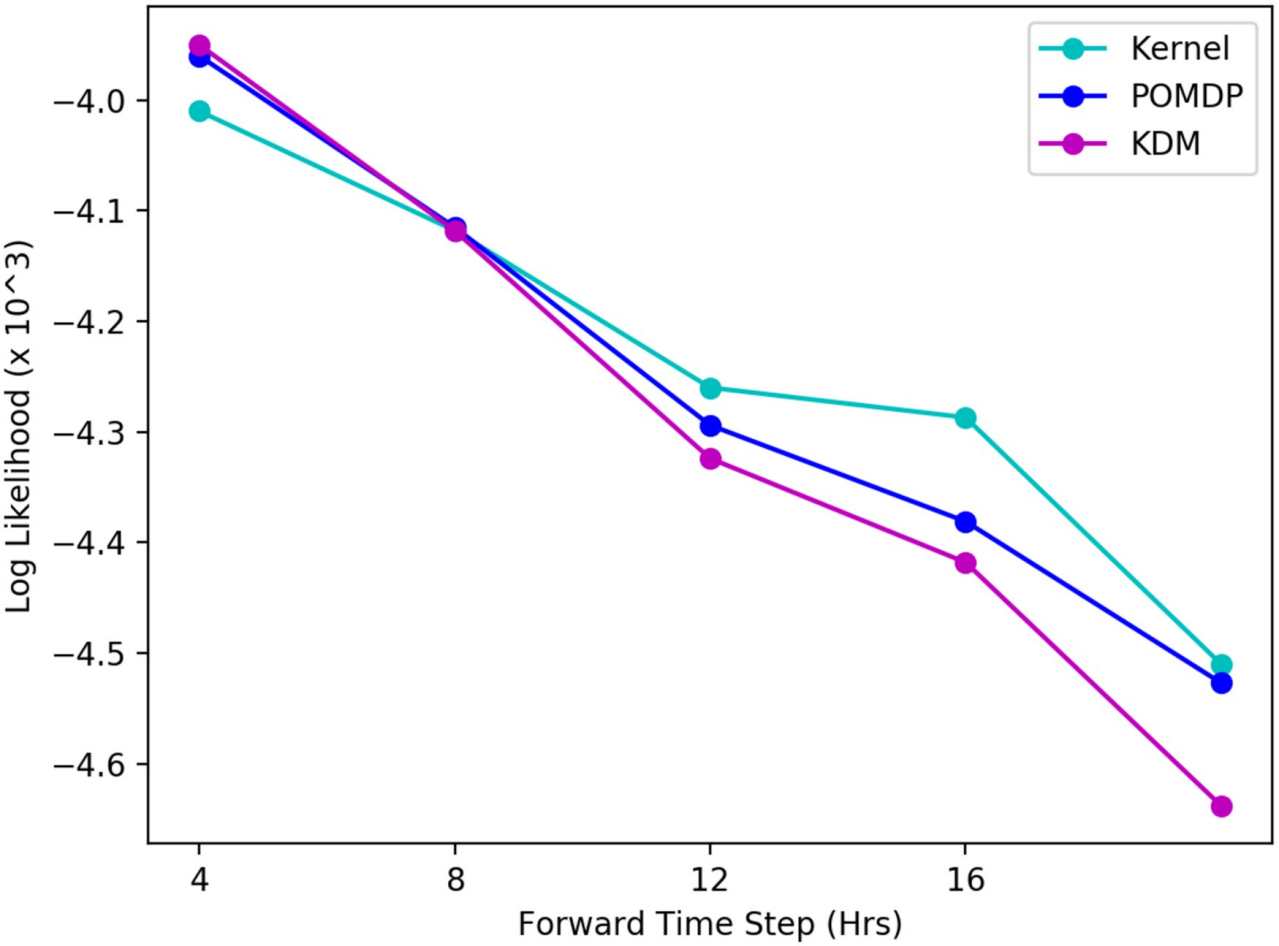
Comparison of predictive log-likelihood across baselines for sepsis for a typical test patient. KDM’s predictions are more accurate across the forward time steps.

In the context of sepsis too, the quantitative differences between the performance of KDM and the MoE policy indicates that the policies are different. As with HIV, we provide an illustrative example. Consider a patient whose blood pressure, heart rate and respiratory rate are all within normal limits. SpO_2_ is used to quantify the saturation of oxygen in the blood. If a patient is initially not ventilated, sedated, or prescribed any vasopressors, we obtain a set of observations and rewards from which subsequent treatments may be selected. Based on the lack of sedation or need to mechanically ventilate initially, KDM predicts the blood oxygen saturation is within normal limits ranging between 90% − 100%. Over the course of 30 hours, this prediction varies marginally when there are minor changes in blood pressure, heart rate and respiratory rate. Throughout this period, no vasopressors are required or prescribed. This is clinically reasonable since vasopressors are typically used to raise the blood pressure hypotensive patients, and are thus not required in this situation. Fig 6 illustrates forward simulating SpO_2_ for the patient described here. The corresponding predictive log-likelihood is shown in Fig 7. As before, the ground truth and respective kernel and POMDP-based predictions are also shown. KDM’s forward predictions are visibly more accurate with respect to the ground truth and contribute to learning a better treatment policy.

**Fig 8:**
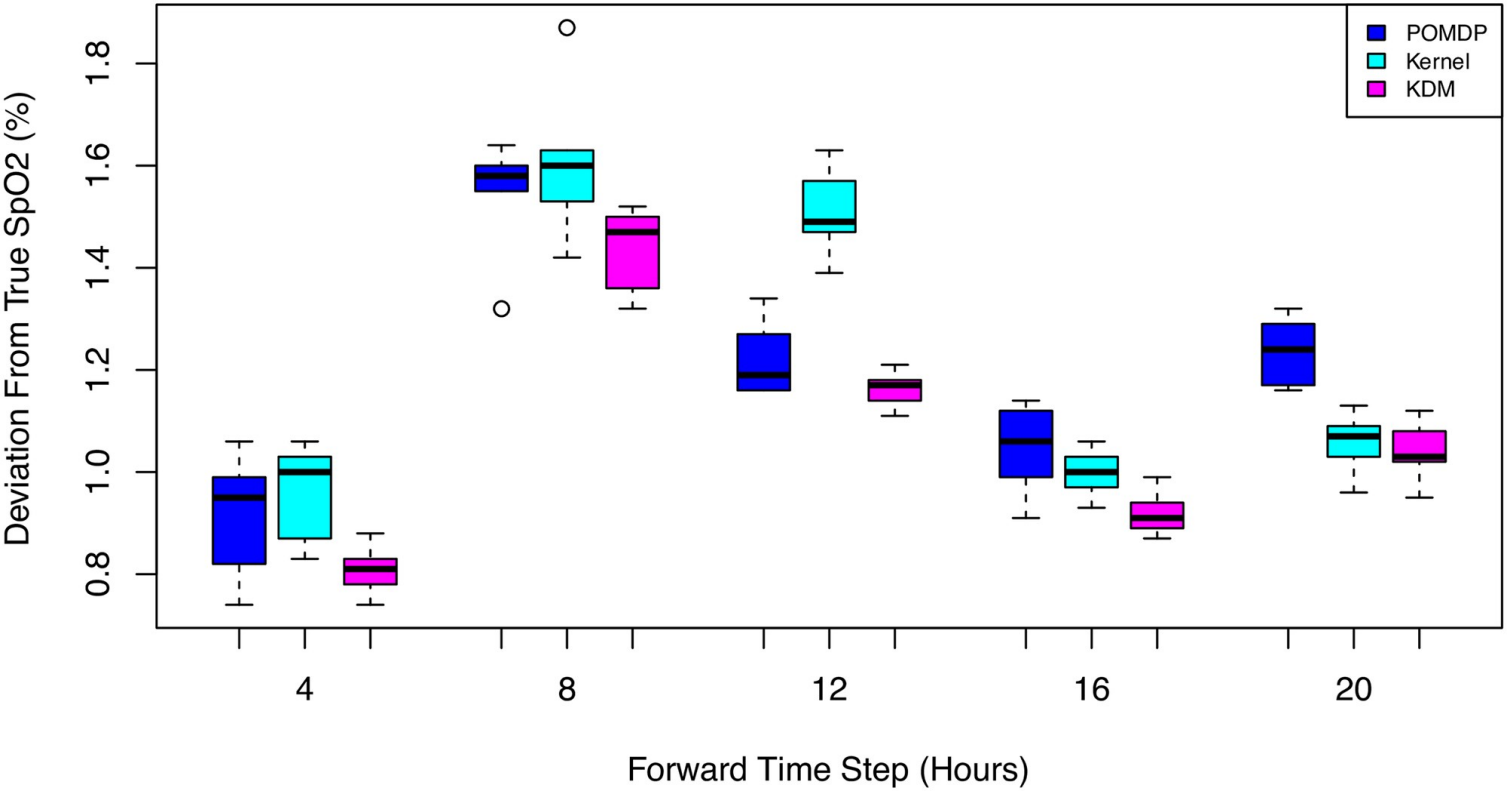
Box plot of SpO_2_ predictions across 3000 test patients under baselines over a 20-hour horizon. KDM’s predictions are closer to the ground truth than POMDP or kernel predictions.

Again, we obtain similar results on the rest of the patients in the test set. Fig 8 illustrates the deviations in counterfactual predictions of SpO_2_ over a 20-hour horizon. KDM is able to model and predict counterfactuals more accurately than the other baselines. This performance is sustained across all time steps.

## 7 Discussion

### KDM produces accurate forward predictions

The KDM policy results in more accurate counterfactual predictions over observation across both the HIV and sepsis tasks. Figs 5 and 8 show the differences at each forward time step between counterfactual predictions using the kernel, POMDP and KDM, and the ground truth across HIV and sepsis patients respectively. Note that these differences cannot be calculated for the MoE policy as this approach does not permit simulating counterfactuals. We observe that across all time steps, the KDM policy tends to predict counterfactuals that are generally closer to the ground truth than those predictions made using the kernel or POMDP methods.

While the kernel and POMDP policies vary considerably over time in their closeness to the true observation, the KDM policy is able to make accurate predictions by combining these predictions and weighting them appropriately.

We can also examine the predictive log-likelihoods of all three approaches for both tasks across each of the forward time steps. An example of these is shown in Fig 4 where we see considerable differences between these values across the methods in the HIV task. For each method, the predictive log-likelihood tends to increase with each forward time step. This is likely a result of more data being available at each successive simulation step in which the histories are grown. Nonetheless, KDM significantly outperforms both the POMDP and kernel approaches at most forward steps. These results are summarised in Tables 6 and 7 for both HIV and sepsis tasks, where we perform a Friedman’s statistical significance test with post-hoc analysis to measure the differences in predictive performance of KDM against the POMDP and the kernel respectively across all test patients. A *p*-value <0.05 here indicates a significant result.

**Table 6:** Friedman’s test measuring predictive performance differences of KDM against POMDP and kernel methods across *t* in HIV. Bold *p*-values correspond to steps where counterfactual predictions from KDM are significantly more accurate than the respective methods. Comparisons with policy-based approaches like MoE cannot be drawn here as these methods cannot be used for counterfactual predictions.

*t*	1	2	3	4	5
POMDP	**0.046**	**0.041**	**0.047**	**0.042**	0.073
Kernel	0.057	0.086	**0.047**	0.058	**0.042**

**Table 7:** Friedman’s test measuring predictive performance differences of KDM against POMDP and kernel methods across *t* in sepsis. Bold *p*-values correspond to steps where counterfactual predictions from KDM are significantly more accurate than the respective methods. Comparisons with policy-based approaches like MoE cannot be drawn here as these methods cannot be used for counterfactual predictions.

*t*	1	2	3	4	5
POMDP	**0.041**	**0.038**	**0.049**	0.083	**0.046**
Kernel	**0.038**	**0.036**	**0.041**	0.091	0.083

### Mixing kernel and model-based RL on a model level produces different policies to mixing on a policy level

Just from the quantitative results, it is clear that the policies produced by our KDM and the MoE are different. We attribute these differences directly to the way in which KDM computes its policy: KDM mixes approaches on the model level, and incorporates these predictions into its belief states for learning an optimal policy. In this way it is able to account for variations across patients at different time points and use these variations to draw new examples of observations from which it can learn. For example in the HIV task, we observe that the KDM policy tends to contain less switches between drug combinations in comparison to the MoE policy. This occurs specifically in cases where patients experience temporary blips or spikes in their viral loads as shown in Fig 3 at 12 and 30 months in the future respectively. Because the KDM policy directly mixes kernel and model based approaches in simulating observations, it can identify these cases more effectively. In these situations, the typical KDM policy does not call for a change in treatments, whereas a MoE policy does. While spurious blips are not regular occurrences, in a clinical setting, it is still important to be able to detect them since it prevents a clinician from potentially exhausting a patient’s future treatment options and exposing them to more potential side effects than necessary.

### KDM leads to interpretable treatment decisions that are clinically face-valid

In both the toy and real experiments, we can demonstrate that the policies obtained using KDM make sense. For the toy task for variants of Type A, KDM correctly chooses *a*_1_ at the second time step, while for variants of Type B, it chooses *a*_2_ here. Since the POMDP is unable to make any informed choice here, the KDM policy typically assigns a higher weight to the nearest neighbour predictions at the second time step and uses these to determine the correct action choice at this step.

For the HIV task, we observe that test patients with higher baseline viral loads tend to sustain higher viral loads and lower CD4^+^ counts in our forward simulations. This is consistent with medical literature that suggests patients with higher baseline viral loads tend to have faster disease progression [38, 39]. In these cases, the KDM policy typically consists of using a nucleoside reverse transcriptase inhibitor (NRTI) such as Zidovudine (AZT), in conjunction with a protease inhibitor (PI) such as Liponavir/ritonavir (LPV/r). Our clinical collaborators confirm that these choices are valid, since a single boosted PI and an NRTI are typically recommended for second-line ART when first-line therapy fails (as indicated by sustaining a viral load above detection limits) [40]. We also checked our treatment policies against current ART guidelines [41, 42]. Overall, we found that our policies were consistent with the recommended first and second-line therapy guidelines 81% of the time. In contrast, the policies obtained from the MoE approach were consistent 76% of the time. KDM policies in violation of IAS-USA recommendations were slightly more likely for patients who started in ART in the early 90s, as standards for combination ART differed significantly at that time. MoE policies in violation of IAS-USA recommendations were more likely for patients experiencing single episodes of low-level viremia or blips, which typically have no clinical consequences, as well as cases where patients were infected by multiple HIV strains. In general, patients infected by multiple HIV strains tend to be more difficult to treat since chances of drug resistance are higher. This, in general, motivates the need for more nuanced treatment policies (e.g. via forward simulation) as suggested by KDM.

There exist less consistent guidelines for the management of fluid and vasopressor administration for patients with sepsis, but we find that the policies recommended by KDM still have many sensible properties, including being consistent with prior work by [36]. The KDM frequently (72% of the time) learn policies where no vasopressors are prescribed. This result is reasonable as vasopressors are used to raise arterial blood pressure in hypotensive patients, and the majority of the test patients do not fall into this category. The KDM policies suggest mechanically ventilating patients with SpO_2_ predictions below 85%, when corresponding predictions of their respiratory rates exceed 29 breaths per second. Several other methods have also been suggested for detecting events such as desaturation and transient hypoxia, but there is frequently a high false alarm rate as described in [43]. In these instances, further clinical expertise is required before intervening. KDM gives us thresholds that we can discuss and debate.

Most importantly, across all three tasks, it is the ability to explicitly step through our forward predictions via KDM that enables us to interpret the policies easily. Overall, we hope that the generative approach of the KDM could help better assess a patient’s overall prognosis and offer more informed therapy choices for intervention.

### The policies obtained from KDM are stable over multiple runs

**Fig 9:**
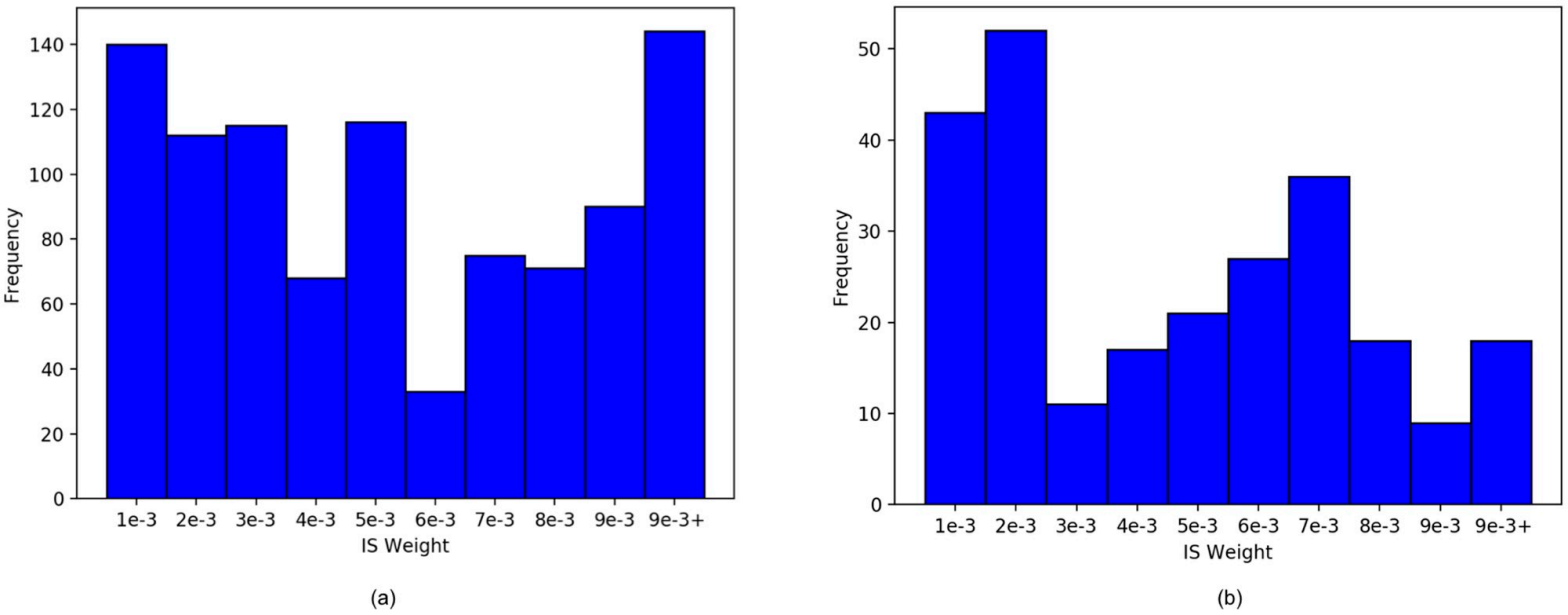
Distributions of frequencies of non-zero IS weights for (a) HIV and (b) sepsis respectively. Our treatments are fairly consistent with those in the data sets.

We tested the performance of KDM over multiple runs on the test data. While the sampled observations and trajectories obtained may differ during forward simulation, the therapy policies obtained across the real world data sets remained virtually identical. Specifically, we obtained fidelity scores of 95% for the HIV domain and 93% for the sepsis task. This stability is crucial to building trust in our policies. A related issue that is frequently encountered when using off-policy evaluation is that only a small fraction of the data contains the treatments suggested by the policies we learn. Fig 9(a) demonstrates that our treatments for HIV are fairly consistent with those in the data set, and at least 1/3 of the test values have non-zero weights. Similar results hold for the sepsis data set in Fig 9(b). This spread is also essential for building trust in our results. That said, these off-policy estimators can be sensitive to the choice of reward and representation; a limitation of all approaches relying on off-policy evaluation is that the reward function is often some surrogate for what we actually wish to optimise, and that we have to assume that the POMDP belief is a sufficient statistic for the history. Developing a more robust form of off-policy evaluation is left as part of future work.

### Limitations and future work

Our results demonstrate that our KDM approach produces a more accurate model (in terms of forward prediction) compared to purely parametric or non-parametric baselines, and using this model for planning produces better policies than either model-based baseline as well as mixing at a policy level. That said, there are still plenty of directions for improving these models: future work could explore alternative ways to design the back-off strategy from kernel to model-based methods (which could themselves be ensembles), and the connections between the regularisation afforded by kPOMDPs or PSRs and our approach. Future work should also develop more accurate off-policy evaluation methods, especially ones that might be robust to the choice of representation.

While creating accurate models is a first step toward building clinical decision support tools, there also exist many steps before methods such as ours are ready to be incorporated into clinical practice. For example, in practice, we may want to ensure that our policies are safe—that is, they never suggest a poor option—rather than simply ensuring higher average performance. Due to the limitations of off-policy evaluation, it would also be important to run any model prospectively to validate the accuracy of the predictions and check if its treatment recommendations are deemed face-valid by clinicians and are in line with clinical guidelines.

## 8 Conclusion

We have introduced kernelised dynamic mixing (KDM), a novel approach for building accurate simulations which mixes between using historical data for predictions when such data are available and falling back on a model when they aren’t. We demonstrate that our KDM approach significantly improves upon policy performance in two real medical tasks—HIV and sepsis management—while also providing the ability to interpret and interrogate the policies via simulating counterfactuals. These steps take us toward being able to provide better decision-support in situations where clinicians must plan over sequences of decisions.

## Supporting information

S1 Appendix Sensitivity to choice of reward functions for HIV therapy selection. Illustration of KDM’s performance relative to varying choices of reward function for the HIV therapy selection task. We tested three alternative formulations of reward functions. Overall, KDM’s performance is relatively robust against the choice of reward function.
(PDF)
