# Supplementary material for: Improving counterfactual reasoning with kernelised dynamic mixing models
Source: PLoS One. 2018 Nov 12;13(11):e0205839. doi: 10.1371/journal.pone.0205839 (PMC6231902; doi:10.1371/journal.pone.0205839)
Supplement: S1 Appendix — Illustration of KDM’s performance relative to varying choices of reward function for the HIV therapy selection task. We tested three alternative formulations of reward functions. Overall, KDM’s performance is relatively robust against the choice of reward function. (PDF) [file pone.0205839.s001.pdf]

Supporting Information Appendix S1

Article title: *Improving counterfactual reasoning with kernelised dynamic mixing models*

Authors: Sonali Parbhoo, Omer Gottesman, Andrew Slavin Ross, Matthieu Komorowski, Aldo Faisal, Isabella Bon, Volker Roth, Finale Doshi-Velez

The following Supporting Information is available for this article:

Sensitivity to choice of reward functions for HIV Therapy Selection

We investigated the performance of the KDM approach against the benchmarks described in the experimentation section with different reward criteria for the HIV therapy selection task. We tested three alternative formulations of reward functions wherein, (a) a higher weight is placed on $CD4^+$ counts than viral load, (b) $CD8^+$ counts are included, (c) a higher weight is placed on the absolute number of mutations than both the $CD4^+$ counts and viral load. These reward functions are given as follows:

(a)

$$r_t = \begin{cases} -0.6 \log V_t + 0.7 \log C_t - 0.2|M_t|, & \text{if } V_t \text{ is above detection} \\ 5 + 0.7 \log C_t - 0.2|M_t|, & \text{if } V_t \text{ is below detection,} \end{cases}$$

(b)

$$r_t = \begin{cases} -0.7 \log V_t + 0.6 \log C_t + 0.6 \log E_t - 0.2|M_t|, & \text{if } V_t \text{ is above detection} \\ 5 + 0.6 \log C_t + 0.6 \log E_t - 0.2|M_t|, & \text{if } V_t \text{ is below detection,} \end{cases}$$

(c)

$$r_t = \begin{cases} -0.7 \log V_t + 0.6 \log C_t - 0.8|M_t|, & \text{if } V_t \text{ is above detection} \\ 5 + 0.6 \log C_t - 0.8|M_t|, & \text{if } V_t \text{ is below detection,} \end{cases}$$

where V_t is the viral load (in copies/mL), C_t is the $CD4^+$ count (in cells/mL), E_t is the CD8+ (cytotoxic T-cell) count (in cells/mL), and $|M_t|$ is the number of mutations at time t.

Our setup was identical to that described in the experimentation section, where the reward criterion was replaced by (a), (b) and (c) respectively. We tested the performance of KDM with the alternative reward criteria on the same held-out set of 3000 patients as before. These results are shown in the following S1 Table A., S1 Table B. and S1 Table C. respectively.

	DR	WIS	IS
Random	–8.42 $\pm$ 2.68	–10.43 $\pm$ 4.17	–10.74 $\pm$ 4.16
Kernel	10.37 $\pm$ 1.71	6.51 $\pm$ 3.86	6.78 $\pm$ 3.60
POMDP	3.57 $\pm$ 1.31	3.82 $\pm$ 2.15	3.68 $\pm$ 2.12
MoE	10.51 $\pm$ 1.20	11.23 $\pm$ 2.10	11.11 $\pm$ 1.99
**KDM**	**12.16 $\pm$ 1.03**	**12.25 $\pm$ 1.20**	**12.38$\pm$ 1.16**

S1 Table A. Performance comparison of KDM vs. baselines for HIV therapy selection across 3000 held-out patients using a POMDP model with 30 states using reward criterion (a). KDM still produces the largest immune response while reducing the viral load, regardless of whether a larger weight is given to CD4$^+$ or V_t.

	DR	WIS	IS
Random	–13.37 $\pm$ 4.17	–12.47 $\pm$ 4.11	–13.61 $\pm$ 4.67
Kernel	12.31 $\pm$ 2.62	10.72 $\pm$ 3.86	12.27 $\pm$ 3.94
POMDP	5.38 $\pm$ 1.15	5.81 $\pm$ 1.46	7.74 $\pm$ 2.51
MoE	13.16$\pm$ 2.60	14.61$\pm$ 1.84	13.84 $\pm$ 2.39
**KDM**	**15.46 $\pm$ 1.10**	**14.81 $\pm$ 1.38**	**16.18 $\pm$ 2.26**

S1 Table B. Performance comparison of KDM vs. baselines for HIV therapy selection across 3000 held-out patients using a POMDP model with 30 states using reward criterion (b). KDM still produces the largest immune response when including cytotoxic T-cell counts E_t in the reward criterion.

	DR	WIS	IS
Random	–12.88 $\pm$ 6.42	–13.65 $\pm$ 7.46	–13.50 $\pm$ 7.16
Kernel	4.65 $\pm$ 4.61	5.28 $\pm$ 4.96	3.73 $\pm$ 6.37
POMDP	1.97 $\pm$ 4.51	4.19 $\pm$ 4.22	**7.18 $\pm$ 4.69**
MoE	4.02 $\pm$ 3.31	**6.29 $\pm$ 3.01**	6.96 $\pm$ 4.81
**KDM**	**6.31 $\pm$ 4.19**	4.55 $\pm$ 3.07	6.31 $\pm$ 3.52

S1 Table C. Performance comparison of KDM vs. baselines for HIV therapy selection across 3000 held-out patients using a POMDP model with 30 states using reward criterion (c). Performance of KDM has significantly higher variance when placing a higher weight on the number of mutations. Evidently in this case, KDM does not always lead to the best immune response.

These results show that the performance of KDM is reasonably robust against the relative weightings of immune response indicators, however placing a heavier negative weight on the ab-

solute number of mutations results in higher variance in the results across all policies, including the other baseline policies. In this case, KDM does not always lead to the best immune response. Since the number of mutations at each point is highly dependent on the number of strains infecting a patient at a time and past exposure to drugs, they can fluctuate considerably across patients. Normalising these counts or incorporating the mutations into a risk score(indicating the likelihood of resistance), rather than including an absolute count in the reward function may overcome this issue. Importantly however, all the methods appear to be sensitive to this choice.
